# Patients' Burden Using Microprocessor-stance-and-swing-control Knee-ankle-foot Orthoses and Outcomes Compared to Those With Prior Traditional Knee-ankle-foot-orthosis

**DOI:** 10.33137/cpoj.v7i1.42799

**Published:** 2024-05-30

**Authors:** B Brüggenjürgen, L Eilers, S Seidinger, A Kannenberg, C. Stukenborg-Colsman

**Affiliations:** 1 Institute for Health Services Research and Technical Orthopedics, Orthopedic Department - Medical School Hannover (MHH) at DIAKOVERE Annastift Hospital, Hannover, Germany.; 2 Otto Bock Healthcare Products GmbH, Vienna, Austria.; 3 Otto Bock Healthcare LP, Austin, Texas, USA.; 4 Foot Department, Orthopedic Department - Medical School Hannover (MHH) at DIAKOVERE Annastift Hospital, Hannover, Germany.

**Keywords:** Knee-Ankle-Foot-Orthosis, Microprocessor-Stance-and-Swing-Control Orthosis, Quality of Life, Activities of Daily Living, Pain, Falls, Orthosis, Disability, Orthotic

## Abstract

**BACKGROUND:**

Patients with neuromuscular knee instability who are fitted with orthotic devices experience issues such as pain, falls, mobility limitations, and restricted participation.

**OBJECTIVES:**

To analyze the burden of disease in patients using a microprocessor-stance-and-swing-control orthosis (MP-SSCO) and, if they had a previous orthosis, to compare their outcomes to those with previous use of a traditional knee-ankle-foot-orthosis (KAFO) under real-world conditions.

**METHODOLOGY:**

A structured cross-sectional survey was conducted in six orthotic and prosthetic clinics in Germany. Individuals who had been using an MP-SSCO (C-Brace) for at least six months, answered an internet-based survey to rate their current and recall previous device outcomes and experience. The questionnaire was self-developed based on established questionnaire design principles and pretested. Patients' well-being dimensions were analyzed with Likert scales. Experiences with current and previous orthotic devices were compared. Falls were analyzed both with and without outliers.

**FINDINGS:**

21 individuals who had used a MP-SSCO for an average duration of two years participated. Fourteen patients had prior experience with a traditional KAFO orthosis. Among them, 78.6% recalled experiencing falls, with a combined annual frequency of 67.9 (SD=167.0, Median=12.0) events. After excluding the two outliers (624 and 182 falls), a mean of 12.1 falls per patient per year was reported (range: 0 to 54, SD=15.9, Median=8.5). With the MP-SSCO, only 42.7% reported falls with an annual frequency of 5.3 (SD=17.0, Median=0.0) falls (p<0.01). After excluding outliers for MP-SSCO users, the average number of falls was 0.5 per year (range 0 to 3, SD=0.9, Median=0.0). This value was significantly lower compared to the previous orthosis (p<0.01). With their previous KAFO, 57.1% of the participants reported being able to walk downstairs, 14.3% to descend stairs with reciprocal gait, and 42.9% to vary their walking speeds. In contrast, 90.5% of MP-SSCO users reported being capable of descending stairs, 81.0% reported to descend stairs with reciprocal gait (p<0.01), and 76.2% claimed they had the ability to walk with varying speeds (p=0.03). Additionally, 71.4% of the respondents experienced an improvement in their engagement in activities with the MP-SSCO. 50.0% reported pain with the previous orthosis, compared to 38.1% with the MP-SSCO. Pain intensity was higher for the previous orthosis use (3.8) compared to MP-SSCO use (2.8) on a 1-5 scale (p=0.06). 93.3% of the participants regarded the MP-SSCO as superior, noting an enhanced quality of life (QoL) compared to the previous orthosis.

**CONCLUSION:**

Advanced orthotic technology may positively impact outcomes such as fall frequency, activities of daily living, fear of falling and pain. However, in this study, results from the previous orthosis use might have been influenced by recall bias.

## INTRODUCTION

Individuals affected by neuromuscular diseases or central nervous system disorders may experience joint instability which restricts their ability to participate in activities of daily living (ADLs). This limitation significantly impacts their engagement in social and professional aspects of life resulting in considerable patient burden. Furthermore, neuromuscular knee instability caused by these conditions can lead to safety concerns for the patients. However, this increased fall risk, could potentially be mitigated by the provision of more advanced and effective orthotic devices.^1,2^

For these patients, orthotic devices designed to stabilize joints, such as ankle-foot orthoses (AFOs) or knee-ankle-foot orthoses (KAFOs), can prove advantageous. AFOs may be sufficient if the knee instability is caused by paresis of the calf muscles. However, if the knee instability is caused by weakness of the quadriceps muscle, AFOs are no longer sufficient. In such cases, knee-ankle-foot orthoses (KAFOs) are frequently utilized to address knee instability associated with neuromuscular diseases and central nervous system disorders.^3^

KAFOs are equipped with knee joints that either lock for both stance and swing phase and are only released manually by the patient when sitting down (locked KAFO) or lock for stance but are free for swing (free-swing KAFOs). Locked KAFOs require substantial compensations to achieve sufficient toe clearance during the swing, such as circumduction, hip hiking, and vaulting.^2,3^ Free-swing KAFOs are further distinguished in posterior-offset KAFOs and stance control orthoses (SCO) that allow the patient to freely swing during the swing phase. These types of orthoses allow for a more natural movement pattern and enable to walk more smoothly on level ground while also providing the needed stability due to locking during the stance phase. Nevertheless, ensuring safe control with these orthoses demands a certain level of remaining motor function in the affected limb. As a result, only a limited proportion of patients can be provided with these systems for reasons of safety.^4^

In the view of experts, the increased risk of falling with both locked and free-swing KAFOs and SCOs contributes to the diminished capacity to walk, which is further impaired by reduced coordination and balance resulting from physical deconditioning.^5^ In addition to the safety concerns associated with traditional orthoses, there are also mobility limitations and restrictions in ADLs as well as pain associated with orthosis use that need to be addressed.^1,6^

Since 2012, a microprocessor stance and swing control orthosis (MP-SSCO) has been available that controls the resistances against knee flexion and extension by hydraulic dampening during weight-bearing, thus enabling knee stance flexion and reciprocal slope and stair descent. It also provides stumble recovery and speed adaptation during the swing phase (Ottobock, Duderstadt, Germany).^7^ Compared to a locked KAFO, an MP-SSCO may facilitate the execution of numerous ADLs by making them easier, more natural, and safer than traditional leg orthosis technologies.^6,8^

MP-SSCOs may allow for walking with increased balance, speed, greater energy efficiency and increased safety.^7^ Objective studies in clinical and lab-based biomechanical settings comparing MP-SSCO to conventional KAFOs have already been performed. User reports emphasized that walking on uneven surfaces, stairs, and ramps was perceived as less challenging, resulting in increased walking autonomy. Independence and mobility are important factors for the daily routine of individuals with lower limb motor impairments and have a decisive influence on their participation and quality of life (QoL).^9^

The burden of disease among leg orthosis users has so far received little attention. Very few studies have focused on demonstrating effectiveness in real-life settings.^1^ Therefore, not only mobility and functionality but also safety and patient burden of disease may be helpful for capturing the indication-specific benefits of a therapy.

According to interviewed experts, patients with muscular knee instability following neuromuscular or central nervous system injuries or conditions who use KAFOs or SCOs experience limited and restricted mobility, impaired gait patterns, and emotional strain.^5^ Advanced orthotic technology, such as the MP-SSCO, might contribute to better QoL of patients, improved gait patterns with subsequent reduction of long-term consequences, and improved perceived dependability and stability of the orthosis while walking.^5^ Nonetheless, there is currently only a limited amount of published data, particularly concerning patient-reported outcomes in an unsupervised home environment. By gathering insights in the patients' perspectives on the MP-SSCO and previous orthoses used, it might be possible to identify unexpected or yet-unknown benefits of the MP-SSCO.

Therefore, the aim of this study was to investigate whether the MP-SSCO provides benefits recognized by patients in real life. A further rationale was to identify whether there were differences to the previous orthoses in patients' reported mobility and safety, satisfaction with the orthotic treatment, and pain. In addition to this assessment, the burden of disease was studied including social environment, aspects of participation in work and ADLs including health-promoting activities, and quality of life.

## METHODOLOGY

A structured cross-sectional survey was conducted in six orthotic and prosthetic clinics in Germany. Included in the study were individuals who met the following criteria: they had been using a C-Brace for more than six months and previously used a traditional locked or free-swing KAFO or SCO, or no orthosis prior to C-Brace fitting. Additionally, the participants had to be older than 18 years of age and provided informed consent. The survey was conducted in the period from December 2020 to November 2021 (seven months during COVID-19 pandemic).

The online tool LymeSurvey was used to send out a link to the German-language questionnaire and collect the responses of participants. Technical reliability was internally tested. Pretest runs of the survey were carried out by different members of our departments. The survey comprised various topics, including demographics mobility/functionality, participation, safety, satisfaction, pain, and quality of life. Questions and response categories were adapted, wherever possible, from existing pain, mobility, and falls questionnaires. The questionnaire employed different types of questions, primarily using Likert scales with 1-5 point rating scales and one time a 1-10 scale for more detailed investigation of general safety perception. Additionally, binary questions and open-response questions were used.

The language of the questionnaire was German. All designers, testers, and participants were native German speakers. Therefore, no translation of the questionnaire was necessary. The responses for the MP-SSCO were concurrent, whereas the responses for the previous orthoses or the state before MP-SSCO fitting had to be recalled from memory. Patients provided informed consent and received a link via email, allowing them to answer the questions at their own pace in a predefined order from the comfort of their homes. The usability of the MP-SSCO was assessed using a summary score that covered various categories and inquired about the patients’ satisfaction with the orthosis in different situations.

The statistical analysis was conducted using IBM SPSS Statistics Version 29.0.0.0. The Wilcoxon one-sided exact test was used for primary comparisons between traditional KAFOs/SCOs and MP-SSCO. Comparisons to traditional KAFOs were performed only in those MP-SSCO users who had experience with such KAFOs (n=14). The Mann-Whitney test was used for sub-analyses. In the case of an extreme range of values, an outlier test was performed using a box plot. Identified outliers were then checked for plausibility. In the case of legitimate outliers, two analyses were performed, one with and one without the outliers, to ensure maximum transparency. Due to the exploratory nature of the study, no sample size calculation was performed.

### Publication Ethics

The observatory investigation is conducted in accordance with the European Medical Device Regulations (Art. 82 MDR), the respective implementation the German Medical Device Law (Section 47 (Paragraph 3) MPDG) and complies with all applicable data protection legislation.^10^

## RESULTS

Out of 22 patients participating in the online survey, which took approximately 60 minutes to complete, 21 participants met the inclusion criteria and were included in the analysis. One participant was excluded due a protocol violation as his previous orthosis had been a first-generation MP-SSCO rather than a traditional KAFO. Participants in the study had utilized the MP-SSCO for a minimum of 11 months (mean 1.9, maximum 2.9 years). Fourteen respondents had prior experience with another KAFO, which they had used for an average of 12.5 years before transitioning to the MP-SSCO. The respondents reported diverse underlying conditions, as indicated in **Table 1**.

**Table 1:** Participant Characteristics.

		N	%
**Age (years)**	All, Mean (SD)=48.1 (14.0); Min=27; Max=71		
	Male, Mean (SD)= 53.6 (12.0)		
	Female, Mean (SD)= 42.1 (14.0)		
**Gender**	Female	10	47.6
	Male	11	52.4
**BMI**	All, Mean (SD)=25.4 (4.0); Min=19.0; Max=33.6		
	Normal weight (BMI 18.5 – 24.9)	11	52.4
	Overweight (25.0 – 29.9)	7	33.3
	Obese (≥ 30)	3	14.3
**Underlying condition**	Poliomyelitis	8	38.1
	Incomplete paraplegia	4	19.1
	Neuromuscular diseases	2	9.5
	Traumatic brain injury (TBI)	1	4.8
	Other	6	28.6
**Comorbidities (multiple responses possible, percentages for those reporting comorbidities)**	None	11	52.4
	Diabetes	1	10.0
	Impaired vision	1	10.0
	Neuropathy	1	10.0
	Total hip replacement	1	10.0
	Hypertension	4	40.0
	Coronary heart disease	1	10.0
	Other	7	70.0
**Affected leg**	Right	6	28.6
	Left	14	66.7
	Both	1	4.8
**Previous Orthosis before MP-SSCO**	Free swing Knee-Ankle-Foot-Orthosis (KAFO/SCO)	10	47.6
	Locked Knee-Ankle-Foot-Orthosis (KAFO)	4	19.0
	No previous orthosis	7	33.3
**Living alone**	Yes	8	38.1
	No	13	61.9
**Independent household management**	Yes	9	42.9
	No	1	4.8
	Partial	11	52.4
**Residential environment**	Flat	10	47.6
	Hilly	8	38.1
	Mountainous	3	14.3
**Residential floor**	0 – ground level	8	38.1
	1^st^	7	33.3
	2^nd^	2	9.5
	3^rd^	1	4.8
	Missing for non-ground level	3	14.3
**Orthosis since (years)**	All, Mean (SD)=12.5 (15.7); Min=1.7; Max=54.5		

Among the 21 survey participants, 10 individuals had additional comorbidities. Out of the 18 respondents aged below 65 years, 14 reported being employed. Moreover, 13 participants were actively involved in more than six ADLs per week, such as shopping, cleaning, gardening, or exercising. Additionally, out of the 13 patients who had to climb stairs to reach their homes, 10 did not have access to an elevator.

Most respondents had used their previous orthoses for more than eight hours per day. 57.1% of the respondents mentioned walking less than 1 km daily, 35.7% walked 1-3 km, and 7.1% reported walking 3-5 km a day with it. Similarly, the MP-SSCO was used for at least 8 hours per day on average. The daily walking distance with the MP-SSCO was reported as less than 1 km by 38.1% of the respondents, 28.6% walked 1-3 km, and 33.3% covered a distance of 3-5 km.

### Safety

The general perception of safety of the prior KAFO (n=14) scored 6.4 on a scale ranging from 1 (completely safe) to 10 (completely unsafe) (**Table 2**). 21.4% of the respondents recalled feeling very safe while standing with the previous orthosis. However, when using the MP-SSCO, the respondents' general perception of safety was rated with an average of 3.8, indicating an improved perception of safety compared to the previous orthosis (p=0.03). Specifically, 76.2% of the respondents felt very safe while standing with the MP-SSCO.

**Table 2:** Comparison of previous orthosis and C-Brace regarding safety, functionality, and pain.

		Mean - previous KAFO (n=14)	Mean – C-Brace in previous KAFO users (n=14)	P-Value	Mean – C-Brace in previous KAFO and non-user (n=21)
**Safety**	General perception of safety^1^	6.4	3.8	0.03	4.1
	Safety while standing^2^	2.8	1.6	0.20	1.4
	Fear of falling^3^	3.4	1.5	<0.001	1.8
	Falls occurred? ^4^	0.8	0.4	0.35	0.4
	Number of falls per year	67.9	1.1	<0.001	5.3
	Number of falls per year, outliers excluded	12.1 (n=12)	0.3 (n=13)	<0.01	0.5 (n=18)
**Mobility & Functionality**	Assistive devices used^4^	0.9	0.6	0.15	0.4
	Usability^5^	3.9	2.0	<0.001	2.1
	Symmetry of gait^5^	4.5	2.1	<0.001	2.2
	Ability to descend stairs^5^	4.0	1. 7	0.01	1.7
	Ability to descend stairs^4^	0.6	0.9	0.06	0.9
	Reciprocal stair descend^4^	0.1	0.8	<0.01	0.8
	Ability to walk an incline^4^	0.5	1.0	0.01	0.9
	Walking at varying walking speeds^4^	0.4	0.8	0.03	0.8
**Pain**	Pain^4^	0.5	0.3	0.08	0.4
	Pain intensity^6^	3.8	2.1	0.06	2.8
	Pain influence^7^	3.7	1.3	0.02	1.7

1 scale ranging from 1 (completely safe) to 10 (completely unsafe)

2 scale ranging from 1 (very safe) to 5 (not safe)

3 scale ranging from 1 (very low) to 5 (very high)

4 no=0/yes=1

5 scale ranging from 1 (very good) to 5 (very poor)

6 scale ranging from 1 (very mild pain) to 5 (very severe pain

7 scale ranging from 1 (no impact) to 5 (severe impact)

With the previous orthosis, the average fear of falling, rated on a scale from 1 (very low) to 5 (very high), was 3.4. However, after switching to the MP-SSCO, the average fear of falling significantly decreased to 1.5 (p<0.001).

Among the users of previous orthoses, nearly 78.6% recalled experiencing falls while using it. On average, these users recalled 67.9 falls per patient (range 0 to 624, Median=12, SD=167.0) over the course of a year. After excluding the two outliers (624 and 182 falls), a mean of 12.1 falls per patient per year was reported (range: 0 to 54, Median=8.5, SD=15.9). After being fitted with an MP-SSCO, only 42.9% of the respondents stated that they had experienced falls in the last six months. The average number of falls with the MP-SSCO was 5.3 per year (range 0 to 78, SD=17.0, Median= 0.0), which was significantly lower compared to the previous orthosis (p<0.001). In patients currently employing MP-SSCO, three outliers of 78 and two occurrences of 12 falls per year were identified. Of note, one user reported experiencing such outliers both with the preceding orthosis and the current MP-SSCO. After excluding outliers for current MP-SSCO users, the average number of falls was 0.5 per year (range 0 to 3, SD=0.9, Median=0.0). This value was significantly lower compared to the previous orthosis (p<0.01). 76.2% of all participants named safety the most critical aspect when assessing the orthosis.

### Mobility & Functionality

Among the respondents who had used traditional KAFOs before, 85.7% mentioned that they had also used additional assistive devices alongside their previous orthoses (**Table 2**). The majority of participants relied on walking sticks or forearm crutches, while 35.7% had used wheelchairs (manual or electric) or scooters, and these devices had been used more than 5 times a week by most. Out of the MP-SSCO users, only 42.9% required other aids such as walking sticks and forearm supports. Furthermore, 35.0% of the respondents had a wheelchair that the majority of them used 5 to 10 times a week.

**Figure 1:**
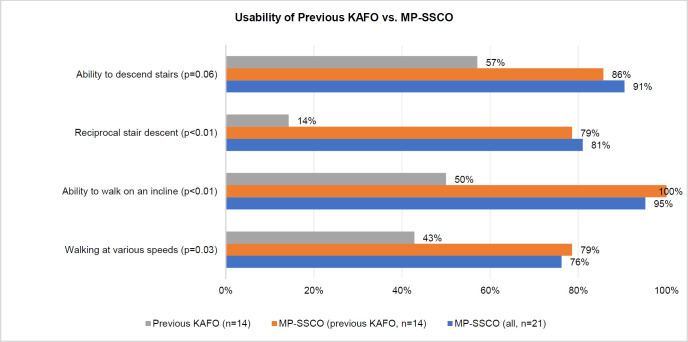
Usability of previous KAFO vs. MP-SSCO separately reported for those with previous experience and all (percentage of participants indicating their capability to perform (yes=1, no=0))

The individuals were asked about their ability to perform various ADLs in their lives including walking at different speeds. In three of the four situations described, there was a significant improvement, while the improvement in one situation just failed to attain significance since using the MP-SSCO (**Figure 1**). The most significant improvement was observed in reciprocal stair descent, with the percentage of participants able to descend a stair with reciprocal gait increasing from 14.3% with the previous orthosis to 81.0% with the MP-SSCO (p<0.01).

Regarding their gait pattern, 92.9% of the participants recalled poor or very poor symmetry with the previous KAFO. However, when using the MP-SSCO, this percentage significantly reduced to only 9.5% (p<0.001). Asked about how they assessed their ability to descend stairs, 75.0% of the patients rated it as bad or very bad with their previous orthosis. However, with the MP-SSCO, 94.7% reported being able to descend stairs well or very well (p=0.01).

**Figure 2:**
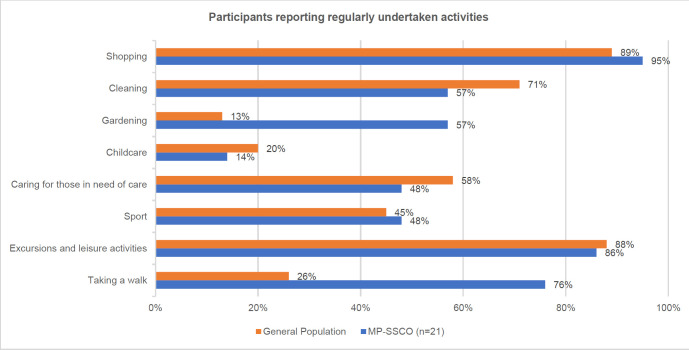
Comparison of physically demanding activities regularly undertaken of participants to the general population: Shopping^11^; Cleaning^12^; Gardening^13^; Childcare^14^ Caring^15^; Sport^16^; Leisure^16^; Walk^17^.

The average usability rating for the previous orthosis was 3.9 (SD=0.6), which indicates poor usability on a scale ranging from 1 (very good) to 5 (very poor). In contrast, the average usability rating for the MP-SSCO was 2.1 (SD=0.7), indicating good usability (p<0.001). Participants being able to pursue physically demanding activities are presented in **Figure 2**.

### Pain

Out of all prior KAFO respondents, 50.0% had experienced discomfort or pain with their previous orthosis, primarily in the form of low-back pain (**Table 2**). On an overall average pain intensity scale from 1 to 5, with 1 representing very mild pain and 5 indicating very severe pain, the average pain intensity was 3.8.

With use and after getting accustomed to the new MP-SSCO, 38.1% of the participants reported pain (p=0.06). The overall average pain intensity for this group was 2.8. The impact of pain on the respondents' daily lives was evaluated using a Likert scale with 1 indicating no impact and 5 indicating severe impact. The average impact of the previous orthosis was 3.7 (quite severe) while that of the MP-SSCO was 1.7 (p=0.02). Those participants engaging in sports reported experiencing significantly more pain (p=0.03).

### Satisfaction with the MP-SSCO & Quality of Life

80.0% of the respondents reported that the C-Brace treatment was considerably superior to their previous orthosis. The satisfaction levels were high, with 71.4% stating they were very satisfied, and an additional 23.8% expressed to be satisfied with the C-Brace. Only 4.7% were not satisfied.

**Figure 3:**
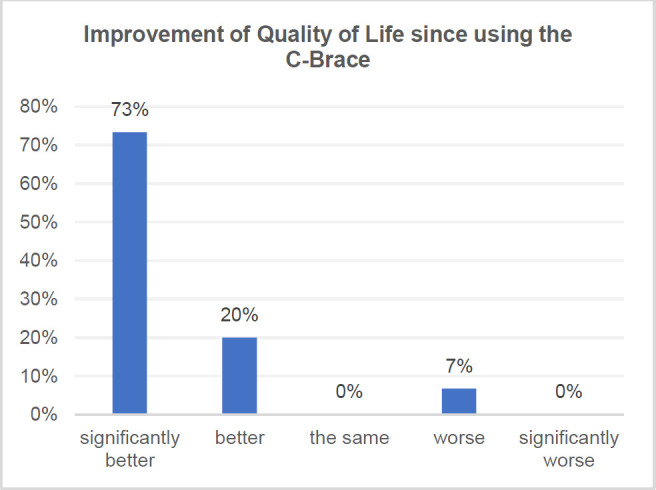
Individuals reporting improvements in quality of life since using the MP-SSCO (n=16).

As shown in **Figure 3**, 15 out of 16 participants who responded on quality of life (QoL) believed that using the C-Brace positively impacted their quality of life. Some patients mentioned specific improvements, such as one individual being able to go skiing and another to start dance lessons. Additionally, many patients appreciated the ability to interact with others at an eye-to-eye level, which had a significant impact on their emotional well-being. When ranking the benefits, the respondents considered safety, effectiveness, and weight as the most crucial factors in their evaluation.

## DISCUSSION

Individuals who had been using an MP-SSCO (C-Brace) for a minimum of six months were invited to participate in an online survey. Twenty-one individuals were included in the analysis. The aim was to gain insights into the real-world impact of conventional locked and free-swing KAFOs or SCOs from the patients' perspective and to understand the differences experienced after using the MP-SSCO. The survey covered various aspects related to safety, mobility, functionality, participation, and pain. The age of the participants varied from 27 to 71 years, and females accounted for 47.6% of the sample, representing relevant user groups for the MP-SSCO in terms of both age and gender.

A major factor that restricts people’s mobility is the inability to negotiate stairs with reciprocal gait. There are still many places that are not accessible to persons with mobility limitations and/or restrictions, and the ability to negotiate stairs is essential for participation.^18^ When rating the ability to descend stairs, 12.5% of the patients using KAFOs recalled it to be good. With the MP-SSCO, 94.7% said they could descend stairs well or very well improving their activities of daily living. Furthermore, more than one third of MP-SSCO users achieved walking distances of the general population - a healthy person in Germany walks approximately 4 km a day.^19^

55% of people with disabilities never engage in sports, while only 33% of people without disabilities do not.^16^ Users of the MP-SSCO demonstrated increased levels of sports activities, with 48% of individuals reporting regular participation in sports. Additionally, when it comes to shopping, excursions, and leisure activities,^11^ the MP-SSCO users achieved levels similar as those of the general population as shown in **Figure 2**. However, our study sample may not accurately reflect the broader population, and there is a lack of published activity data with sufficient detail to enable a more precise comparison within the studied populations. Consequently, our evaluation sought to determine if individuals equipped with the MP-SSCO experienced significant limitations in their daily activities.

Given the considerable personal commitment and potential financial investment required for orthotic provision, it is presumed that the subset of individuals using the MP-SSCO represents an active and potentially affluent subgroup seeking to maximize their newfound opportunities. Consequently, the notably higher prevalence of gardening and walking activities could be attributed to two factors. Firstly, a higher proportion of MP-SSCO users may have access to private yards or gardens. Secondly, walking may be perceived as a privilege or preferred form of exercise compared to other physical activities such as jogging.

The use of MP-SSCOs resulted in a reduction in the number of individuals who recalled pain, a decrease in average pain intensity, and a significant reduction in limitations in their ADLs. It is important to consider that the increased activity levels of patients since using the MP-SSCO may have influenced the observed differences. Interestingly, when comparing MP-SSCO users who exercise with those who do not, the ones who exercise tended to report more pain on average. Despite the presence of some pain while using the MP-SSCO, it is perceived as significantly reduced in intensity and less restrictive in ADLs. The majority of patients expressed satisfaction with the C-Brace, which was evident in its significantly improved usability score compared to the previous orthosis.

The conditions leading to the use of an MP-SSCO in this patient survey were comparable to those mentioned in interviews with experts.^5^ According to the experts interviewed, the primary patient burdens leading to the use of a MP-SSCO were “limited mobility,” followed by “emotional stress” and “altered gait pattern.” The most significant factors affecting patients were identified as the dependability and stability while walking, restoring a natural gait, and the ability to perform ADLs.^5^

Our patient survey revealed that safety and effectiveness were considered as the leading relevant aspects, and that aligns with the views expressed by the experts. However, the weight of the orthosis emerged as the third-most relevant aspect, which was not explicitly highlighted by the experts.

A health-technology assessment (HTA) and systematic patient survey identified the reduction of pain, falls, and the improved ability to perform ADLs as the outcomes primarily desired by patients, while the most valued orthosis features were reported to be effectiveness, reliability, comfort, and durability, as presented in **Table 3**. However, the existing evidence on the effectiveness of orthoses is limited, especially when focusing on the most important outcomes from the perspective of the users.^1^

**Table 3:** Comparison of leading aspects from different survey sources.

	Patient survey	Expert survey^5^	O´Connor analysis^1^
Important aspects		Perception of safety and high stability while walking Physiologic gait Participation in daily life	Engagement in daily activity
Patients desired outcomes	Safety Independence Quality of life Meeting fellow people on eye level Improved gait	Reliability Stability while walking Physiologic gait Ability to perform daily routine (data on file) Improved quality of life	Reduction in pain, falls or trips. Improved balance and stability
Most valued features	Effectiveness Safety Weight	Improved quality of life Improved gait patterns High dependability of the orthosis	Effectiveness Reliability Comfort and durability
Frustration	Uncomfortable (n=1) and not water proof (n=1)	Setting Correct indication Intensive service support Access to reimbursement decision maker	Perceived deficiencies in service provision related to: Appointment Administrative systems and Referral pathways
Lead patient burden		Restriction of mobility, emotiona strain, impaired gait pattern	
Other burden		Appearance and good look Cosmetic issues	Appearance - Want to look as normal as possible. Women are especially affected in dressing

Among the individuals in our patient survey, 76.2% regarded safety as the most critical aspect when assessing the orthosis. In contrast, the experts prioritized dependability and stability, and they did not consider safety to be as significant a factor when evaluating an orthosis.^5^

With the MP-SSCO compared to traditional KAFOs, the average number of falls per year was reduced significantly by 92.2%. It is noteworthy that the presence of extreme outliers in the survey may have influenced these averages. As per the expert interviews, falls occurred in 71.5% of patients using KAFOs or SCOs with a combined annual frequency of 7.0 fall events. In contrast, the experts had observed falls in only 7.2% of MP-SSCO users and an annual frequency of 2.2 fall events per year. This suggests that the use of MP-SSCO resulted in a considerable reduction in fall incidents compared to traditional types of orthoses.^5^ However, the numbers also suggest that patient may not report all falls to their treating physicians.

According to the experts, advanced orthotic devices may enhance physical and psychological health and well-being by enabling patients to pursue their daily routines.^5^ This was directly confirmed by patients’ results in our study regarding usability and improved quality of life. Improved gait patterns were not explicitly mentioned by patients but might result in subsequent reduction of long-term consequences, according to experts.^5^

Experts' and patients' perspectives might differ. Horenkamp et al.^20^ investigated the impact of obtaining results from different perspectives. Experts’ estimate on resource use or patients’ burden was principally lower when compared to patients’ own reported outcomes. Hence, this is in line with the comparative results reported in our analysis.^20^

Our results confirm the need for incorporating outcomes that are relevant to the patients into research of orthotic devices for knee instability related to neuromuscular and central nervous system conditions.

### Study limitations

The retrospective nature of this study results in a possible recall bias. Recall bias refers to different results in interviews or self-reports of previous exposures or events. It is therefore primarily a problem in retrospective studies. This collection of past information, especially on the situation before the current MP-SSCO and when actively using it, carries the risk of recall bias as a form of information bias when collecting retrospective data. In general, patients tend to overestimate pain and underestimate mobility in the past.^21^ Additionally, there could be a technology bias since all patients were already using the MP-SSCO at the time of the survey, especially affecting previous KAFO results. This refers to preconceived positive notions of the used technology and hence biased opinions in favor of adopting the technology.^22^ Another consequence arises from the number of individuals who participated in this study, potentially leading to a bias due to a greater influence of extreme values. Finally, though outcome measures in this exploratory study were developed according to sound empirical practices and applied standard response items such as Likert-scales, a prior validation had not been conducted.

## CONCLUSION

Individuals with knee instability caused by neuromuscular diseases or central nervous system disorders face limitations and restrictions in mobility, impaired gait patterns, and experience emotional strain when using conventional locked and free-swing KAFOs. The implementation of advanced orthotic technology has the potential to positively impact patient-specific parameters such as fall frequency, fear of falling, pain, activities of daily living, and reliance on other assistive devices. Notably, the use of MP-SSCO resulted in a significant reduction in the frequency of falls compared to conventional KAFOs.

When assessing their orthoses, individuals emphasized safety, effectiveness, and weight as the most relevant attributes. In contrast, experts considered dependability and stability as the most crucial aspects. This highlights the importance of involving patients in the orthosis selection process and taking into account the aspects that users rate as most significant. Furthermore, individuals' quality of life was identified as a relevant dimension and an area that could see substantial improvements for KAFOs/SCOs users.

## DECLARATION OF CONFLICTING INTERESTS

**Bernd Brüggenjürgen:** Received lecture fees.

**Lena Eilers:** Nothing to be declared.

**Susanne Seidinger:** Full-time employee of Ottobock.

**Andreas Kannenberg:** Full-time employee of Ottobock.

**Christina Stukenborg-Colsman:** Nothing to be declared.

## AUTHORS CONTRIBUTION

**Bernd Brüggenjürgen:** Conception and design, analysis and drafting of the paper, interpretation of the data; revising it critically for intellectual content and final approval of the version to be published.

**Lena Eilers:** Analysis and drafting of the paper, interpretation of the data; revising it critically for intellectual content.

**Susanne Seidinger:** Conception and design, interpretation of the data; revising it critically for intellectual content and final approval of the version to be published.

**Andreas Kannenberg:** Interpretation of the data; revising it critically for intellectual content and final approval of the version to be published.

**Christina Stukenborg-Colsman:** Interpretation of the data; revising the manuscript critically for intellectual content and final approval of the version to be published.

## SOURCES OF SUPPORT

This work was supported by an unrestricted grant of OttoBock Healthcare Products GmbH, Wien.
